# Oral and Dental Section of the Ninth International Medical Congress

**Published:** 1886-01

**Authors:** 


ORAL AND DENTAL SECTION OF THE NINTH INTERNATIONAL MEDICAL CONGRESS.—So far as we have observed, the journals in different parts of the country devoted to the interests of oral and dental science and practice, give expressions of cordial approval of the present preliminary organization of the Congress, and especially of the organization of the Section of Oral and Dental Surgery. The only exception we have noticed is that of the *Independent Practitioner*, published at Buffalo, whose editor, in the December number, has the following singular statement: "Following this (the meeting of the Committee on Organization, in Chicago, in June last) came the announcement that the Section of Dental and Oral Surgery was, by the reorganized Committee, abolished, and that dentists would not be welcomed to the Congress, upon the ground that such medical men as had devoted their lives to the consideration of diseases of the oral cavity were not engaged in the legitimate practice of any branch of the healing art."

It is a sufficient answer to all this, to simply state the fact that no such announcement was ever authorized by the "reorganized Committee," nor by any other party representing the American Medical Association· On the contrary, the latter body had fully recognized the relations of dental and oral surgery to the general field of medicine and surgery several years since by organizing and maintaining a Section in that department on the same level with all its other Sections. The simple facts are that the Committee of Arrangements at its first meeting in June found nineteen Sections organized, which it was then thought was a greater number than could be accommodated with convenient rooms in Washington, and more than had been provided for at any of the preceding Congresses. Solely for the purpose of reducing the number, propositions were made and temporarily adopted to discontinue the Sections of Mental and Nervous Diseases, of Gynecology, and of Dental ond Oral Surgery, leaving the workers in those departments to take their places in the Sections of General Medicine, Obstetrics, and General Surgery, respectively. We say temporarily adopted, because neither the revision of the Rules nor of the Sections was completed and finally adopted by the Committee until its second meeting, in September, when, after more consideration, all the three Sections, the discontinuance of which had been proposed, were retained without a dissenting vote in the Committee; that of Dental and Oral Surgery without the change of a single officer. And there is every indication that the Section will be sustained with a degree of enthusiasm that will not only do credit to the many scientific workers in that department, but will also aid much in restoring that department to its proper place, as a legitimate branch of the healing art, much to the benefit of both specialists and general practitioners; and it is to be hoped that this Section will be made so eminently successful that no Congress hereafter will be considered complete without it; for we agree fully with the renowned Virchow, that no one department can be isolated altogether without detriment to the whole.—*Journal of American Medical Association.*

